# The Role of Nephronectin on Proliferation and Differentiation in Human Dental Pulp Stem Cells

**DOI:** 10.1155/2017/2546261

**Published:** 2017-11-19

**Authors:** Jia Tang, Takashi Saito

**Affiliations:** ^1^Division of Biochemistry, Department of Oral Biology, School of Dentistry, Health Sciences University of Hokkaido, 1757 Kanazawa, Ishikari-Tobetsu, Hokkaido 061-0293, Japan; ^2^Division of Clinical Cariology and Endodontology, Department of Oral Rehabilitation, School of Dentistry, Health Sciences University of Hokkaido, 1757 Kanazawa, Ishikari-Tobetsu, Hokkaido 061-0293, Japan

## Abstract

**Aim:**

The purpose of the current study was to investigate the effects of nephronectin (Npnt) in human dental pulp stem cells (hDPSCs).

**Methodology:**

Npnt was coated to nontissue culture-treated polystyrene (non-PS) plates. The presence of immobilized protein on the surface was detected by polyclonal rabbit primary anti-Npnt antibody. Then the cell number was counted and compared with PBS-, bovine serum albumin- (BSA-), fish scale type I collagen- (FCOL1-), and human fibronectin- (Fn-) coated wells. Cell proliferation was assessed using CCK-8 assay. Cell morphology was observed under light microscopy and fluorescence microscopy. Lastly, the mRNA expression profiles of integrins, dentin sialophosphoprotein (DSPP), bone sialoprotein (BSP), and mineralization capacity of hDPSCs were investigated by real time RT-PCR and alizarin red staining, respectively.

**Results:**

Npnt mediates hDPSC adhesion and spreading partially via the Arg-Gly-Asp (RGD) motif. Npnt enhanced the mRNA expression of ITGA1, ITGA4, ITGA7, and ITGB1 on day five. Npnt downregulated DSPP but significantly upregulated BSP mRNA expression at day 28. Further, Npnt and FCOL1 accelerated the matrix mineralization in hDPSCs.

**Conclusions:**

The current findings implicate that Npnt would be favorable to recruit hDPSCs and conducive to mineralization in hDPSCs. The combination of Npnt with hDPSCs may offer a promising approach for hard tissue regeneration.

## 1. Introduction

A healthy dental pulp is of paramount importance to the structural and functional integrity of the tooth. The implications caused by tooth devitalization such as discoloration have driven significant interest in the development of bioactive materials that facilitate the regeneration of damaged dentine tissues by harnessing the capacity of dental pulp for self-repair.

As the regeneration capacities of dental tissue are limited, exposure of pulp, whether caused by injury or caries, requires immediate operative intervention to restore its vitality. The fundamental point of a successful regeneration of dentin is a combination of growth factors, stem cells, and scaffold. The human dental pulp stem cells (hDPSCs) were discovered in 2000 and characterized by their ability to form dentine-like structure when transplanted into immunocompromised mice [1]. On the other hand, hDPSCs were also able to differentiate into adipocyte and neural-like cells [2], highlighting their potentiality to be used in the treatment of neurodegenerative diseases. Recently, it was reported that hDPSC secretome reduces cytotoxicity and apoptosis caused by amyloid beta (A*β*) peptide, a main component of amyloid plaques in Alzheimer's disease (AD), and could possibly be utilized in the treatment of AD [3], a chronic neurodegenerative disease that afflicts 46 million people worldwide nowadays [4]. More importantly, although DPSC shares similar immune-phenotype with the bone marrow stromal cell (BMSC) *in vitro*, DPSC displayed strikingly higher odontogenic potentiality than BMSC using the same induction factors [5].

Generally, because of the existence of stem cells, tissues or organs retain an innate ability to repair their damaged portions, to a certain degree. Nevertheless, this endogenous capacity is compromised by aging or severe inflammation. Tissue engineering in the dental field is therefore gaining an increasing interest. The past several decades witness a paradigm shift in the conception of the scaffold, which has changed from passive carrier to bioactive signal initiator. Especially, relentless efforts have been ongoing in exploring natural polymers such as extracellular matrix (ECM) scaffold containing cell interactive motifs. To name a few, collagen [6], chitosan [7], hydroxyapatite [8], etc. have all been reported to be appropriate candidates for tissue engineering. Npnt, an Arg-Gly-Asp- (RGD-) containing ECM protein originally identified in embryonic kidney, is intensively expressed in developing tooth and assumes important function in regulating the sox2 expression, a dental epithelial stem cell marker. Importantly, loss of Npnt results in reduced tooth germ size [9], underscoring an indispensable role of Npnt in the process of tooth development. Furthermore, an earlier work from our lab demonstrated that the mRNA expression of Npnt increased with the differentiation of the MDPC-23 cell and Npnt protein itself was effective in promoting the proliferation and differentiation of this specific odontoblast-like cell line [10]. Nevertheless, the precommitted nature of MDPC-23 cell necessitates study using a multipotent type of cell to further confirm the adhesive and inducing capacities of Npnt.

The current experiment was hence designed to clarify the following issues: first, is Npnt a hDPSC adhesive? Second, if yes, is RGD in its sequence involved in the regulation of cell adhesion? Third, is Npnt effective in promoting the differentiation of hDPSCs into odontoblast or at least hard tissue-forming cells? Finally, can the mineralization of hDPSCs be enhanced by Npnt? To answer those questions, we coated Npnt protein to nontreated tissue-cultured polystyrene plates (non-PS) and assessed a number of biochemical parameters such as RGD peptide inhibition assay, CCK-8 assay, real-time RT-PCR, and alizarin red staining to characterize the cellular behavior.

## 2. Materials and Methods

### 2.1. Cell Culture

Human dental pulp stem cells (hDPSCs) (catalog number PT-5025; Lot number 0000361427) were purchased from LONZA (Walkersville, MD, USA). Use of hDPSCs was approved and under the guidelines set by the ethical committee of the Health Sciences University of Hokkaido. The characterization of its stem cell phenotypes was carried out using flow cytometry including tests of surface antigens (CD105^+^, CD166^+^, CD29^+^, CD90^+^, CD73^+^, CD133^−^, CD34^−^, and CD45^−^) by the manufacturer (see the certificate of analysis in Supplementary file 1 available online at https://doi.org/10.1155/2017/2546261). Cells were maintained in Dulbecco's Modified Eagle's Medium (DMEM) (D5796, Sigma) supplemented with 10% fetal bovine serum (FBS) (10270-106, Gibco). All media were supplemented with 50 units/mL penicillin and 50 *μ*g/mL streptomycin (catalog number 15070063, Gibco). Cells were cultivated at 37°C under humidified 5% CO_2_ and 95% air atmospheric conditions. Mineralization reagent including *β*-glycerophosphate (*β*-GP, 10 mM) (191-02042, Wako) and ascorbic acid (AA, 50 *μ*g/mL) (013-19641, Wako) was incorporated upon confluence.

### 2.2. ECM Proteins and Coating Procedure

Non-PS plates (24-well plate: 1820-024, Iwaki; 12-well plate: 351143, Falcon) were coated with nephronectin (Npnt, 10 *μ*g/mL, 1 *μ*g/cm^2^, 4298-NP-050, R&D systems), fibronectin (Fn, human plasma, 10 *μ*g/mL, 1 *μ*g/cm^2^, 33016015, Gibco), and fish scale type I collagen (FCOL1, 10 *μ*g/mL, 1 *μ*g/cm^2^, Cellcampus AQ-3LE, Taki Chemical) diluted in phosphate-buffered saline (PBS) (REF 10010-023, Gibco), water (pH 7.4), or sterile acidic water (pH 3.0), respectively. PBS- and bovine serum albumin (BSA, 1%) (A9418, Sigma)-coated substrate(s) were served as controls. Surfaces were coated with ECM proteins solution for 48 hours at room temperature inside clean bench, washed twice with PBS before cell inoculation.

### 2.3. Confirmation of the Presence of Coated Npnt by Immunofluorescence Staining

Primary polyclonal rabbit anti-Npnt (1 *μ*g/mL, ab110230, Abcam) was added to Npnt- or PBS-coated polystyrene at room temperature for 2 h. PBS was used to wash the first antibody-treated surfaces twice. Afterwards, the surfaces were incubated for 1 h in PBS with Alex Flour 488 goat anti-rabbit IgG (2 *μ*g/mL, A11034, Invitrogen). Surfaces were rinsed twice by PBS and imaged using fluorescence microscopy (EVOS® FL Cell Imaging System, catalog number AMF4300, Thermo Fisher Scientific).

### 2.4. Fluorescence Staining of Actin Cytoskeleton

hDPSCs were seeded into 24-well plates (surface area: 2 cm^2^, non-PS) at the concentration of 4 × 10^3^/well in DMEM containing 10% FBS. At day five, culture media were aspirated and cell monolayer was rinsed by PBS (Gibco) twice preceding fixation. Fixation of cells was performed using methanol-free formaldehyde (16%, *w*/*v*, catalog number 28906, Thermo Fisher Scientific) diluted to a concentration of 4% (*v*/*v*) in PBS (200 *μ*L/well) for 15 min at room temperature. Cells were rinsed briefly by PBS three times before addition of permeabilisation reagent Triton X-100 (0.1%, *v*/*v*, in PBS) (400 *μ*L/well). Five minutes following the permeabilisation treatment, BSA at the concentration of 1% (*w*/*v*, in PBS) was poured into each well (400 *μ*L/well) to block any nonspecific binding for 30 min at room temperature. Phalloidin (catalog number A12380, Invitrogen) (working concentration: 2 U/200 *μ*L, 200 *μ*L/well), a highly toxic and specific cytoskeleton probe conjugated to red-orange fluorescent Alexa Fluor® (AF) 568 dye, was used to localize F-actin. Nucleus was counterstained using 4,6-diamidino-2-phenylindole (DAPI, D9542, Sigma) (working concentration: 300 nM, 200 *μ*L/well) for 5 min. Finally, cells were immersed in PBS to avoid drying up and photographed using EVOS FL Cell Imaging Station System. Four different fields were selected under fluorescence microscopy to count the number of DAPI-stained nucleus. The average number was calculated and compared between each group.

### 2.5. Cell Proliferation Assay

For the cell viability test, fifty microliters of recombinant Npnt (10 *μ*g/mL) solution was coated onto a 96-well plate (351172, Falcon) for two days at room temperature. The wells were dried up and rinsed twice with PBS. hDPSCs were rinsed once with PBS, harvested using trypsin for eight minutes at 37°C, collected by centrifugation (500*g*, 5 min, 2800, Kubota), and then resuspended in serum-free DMEM. Cells were seeded into non-, BSA-, Npnt-, Fn- and FCOL1-coated wells at the concentration of 8 × 10^3^/well. At 18 h, 41 h, and 64 h, CCK-8 reagent was added to each well (10 *μ*L/well) and incubated for 1 h and 20 min in the incubator. Absorbance was read at the wavelength of 450 nm (iMark™, Bio-Rad).

### 2.6. Inhibition Test Using Npnt-Derived Soluble RGD Peptide

The RGD-containing hexapeptide, KPRGDV, was designed based on the amino acid sequence of mouse Npnt (NCBI reference sequence NP_001025007.1). KPRGDV and its aberrant counterpart KPRGEV with final purity over 97% were generated from Sigma-Aldrich, Japan. The peptides were dissolved in PBS to a final concentration of 10 mM and stored at 4°C until use. Npnt (10 *μ*g/mL) solution was poured into a 24-well plate (200 *μ*L/well), and the plate was dried up with a cover open in the clean bench for two days. Prior to cell inoculation, the coated plate was rinsed briefly with PBS twice (400 *μ*L/well each time). hDPSCs were preincubated with KPRGDV (1 mM) or KPRGEV (1 mM) or PBS vehicle for 10 min at 37°C in the incubator. After incubation, the cells were seeded into Npnt-coated wells at the concentration of 2 × 10^4^/well in serum-depleted DMEM. The cell photos were taken after 19 h incubation; afterwards, the media were changed into DMEM containing 10% FBS. Cells were photographed at 43 h and 67 h as well.

### 2.7. Quantitative Reverse Transcriptase Polymerase Chain Reaction (qRT-PCR)

hDPSCs were seeded into 12-well plates coated by either Npnt or the other proteins (BSA, Fn, and COL-1) at the concentration of 2 × 10^4^/well and incubated at 37°C in 5% CO_2_. After five and 28 days in culture, cells were lysed by TRIzol (Invitrogen) and total RNA extracted from each sample using the acid guanidinium thiocyanate-phenol-chloroform method. After the precipitation step, RNA was washed with 75% ethanol and reconstituted in RNase-free water. Quantification of RNA was measured by absorbance using Nanodrop 1000 (Thermo Fisher Scientific). Total RNA was reverse transcribed into complementary DNA (cDNA) and amplified in a 20 *μ*L reaction system. Real-time RT-PCR was carried out in LightCycler® Nano (Roche) using FastStart Essential DNA Probes Master (2x) (Roche). Housekeeping gene GAPDH was taken to be the internal control. The primers used are shown in Table 1. The SYBR green amplification consisted in an initial denaturation of 10 min at 95°C and followed by 50 cycles of 15 s at 95°C (denaturation), 30 s at annealing temperature (refer to Table 1 for each set of primer) and 40s at 72°C (extension).

### 2.8. Alizarin Red Staining (ARS)

ARS is a method of detecting calcium-rich deposits in cell cultures. The media were aspirated before the staining procedure, cells were rinsed briefly with PBS twice and fixed by adding 10% neutral buffered formalin (200 *μ*L/well in a 24-well plate; 400 *μ*L/well in a 12-well plate) (060-01667, Wako) at room temperature for 20 min. Fixative residues were removed by washing with distilled water. ARS solution (1%, pH 4.0) (011-01192, Wako) was added, at the same volume of fixative poured, and incubated at 37°C for 5–10 min. ARS solution was discarded afterwards; the cells were washed using distilled water for another 1 hour and photographed. To quantify the staining intensity, cetylpyridinium chloride (CPC) (10%, *w*/*v*, in distilled water) (C0732-100G, Sigma) was added to each well (700 *μ*L/well) to extract the stain. After 1 h incubation under 37°C, the CPC solution was transferred to a new 96-well plate (200 *μ*L/well) for absorbance reading at 570 nm.

### 2.9. Statistical Analysis

Statistical analyses were performed by post hoc Tukey's HSD test. The results were considered statistically significant for *p* < 0.05.

## 3. Results

### 3.1. Confirmation of the Presence of Npnt on Polystyrene Surface

**Figure 1:**
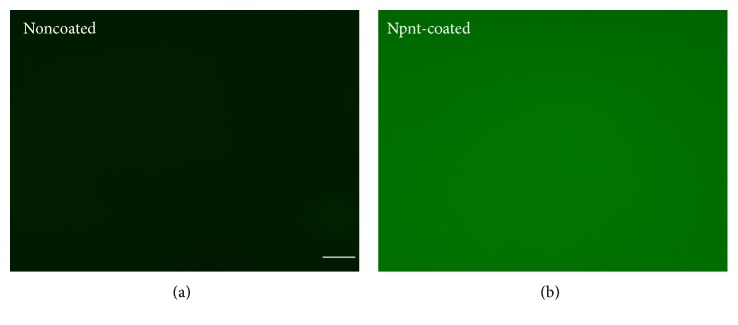
Fluorescence visualization of coated Npnt. Npnt-coated polystyrene was positive for anti-Npnt (b). No fluorescence was observed in the noncoated control (a) (scale bar: 50 *μ*m).

The Npnt-coated polystyrene was positive for anti-Npnt-generating green fluorescence light, while no fluorescence was observed in the PBS-coated control (Figure 1).

### 3.2. Cell Morphology Observation and Cell Number on Different ECMs

**Figure 2:**
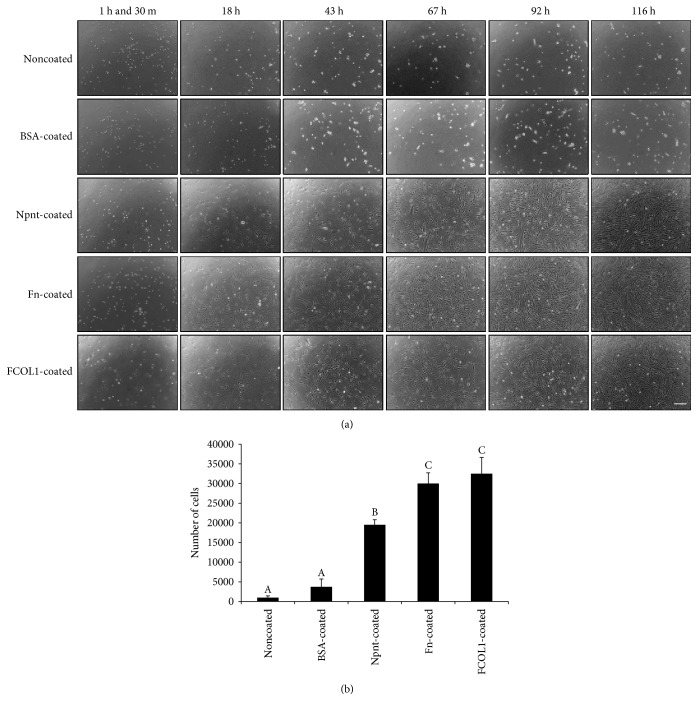
Microscopic observation and cell number counting. (a) hDPSCs were seeded into PBS-, BSA-, Npnt-, Fn-, or FCOL1-coated 24-well plates (non-PS) at the concentration of 4 × 10^3^/well in DMEM supplemented with 10% FBS, penicillin/streptomycin (50 U/mL; 50 *μ*g/mL) (scale bar: 200 *μ*m). Cell morphology was observed at 1 h and 30 m, 18 h, 43 h, 67 h, 92 h, and 116 h. (b) hDPSCs were seeded into 24-well plates (non-PS) at the concentration of 4 × 10^3^/well in the same culture media as illustrated in (a), cell number was counted at day five. Different symbols represent significant differences, *p* < 0.01 by post hoc Tukey's HSD test.

The light microscopy photos of cells seeded on five different substrate(s) are shown in Figure 2(a). Until 116 h, it was observed that cells in noncoated and BSA-coated groups remain to be round in shape, no cells were found to be spread in the two groups. Cells in Npnt-, Fn-, and FCOL1-modified surfaces were successfully attached, spread, and adopted-flattened or spindle-shaped; the cells continued to grow during the five-time points of observation in the three groups. A significant difference in cell number was observed in Npnt- (1.95 ± 0.13 × 10^4^), Fn- (3.00 ± 0.27 × 10^4^), and FCOL1- (3.25 ± 0.41 × 10^4^) coated substrate(s) as compared to PBS- (0.10 ± 0.04 × 10^4^) and BSA- (0.38 ± 0.20 × 10^4^) coated control groups at day five. The cell number in the Npnt-coated group was slightly lower than that in Fn- and FCOL1-coated groups (*p* < 0.01), while no difference was detected between Fn- and FCOL1-coated surfaces (Figure 2(b)).

### 3.3. Cytoskeleton Visualization

**Figure 3:**
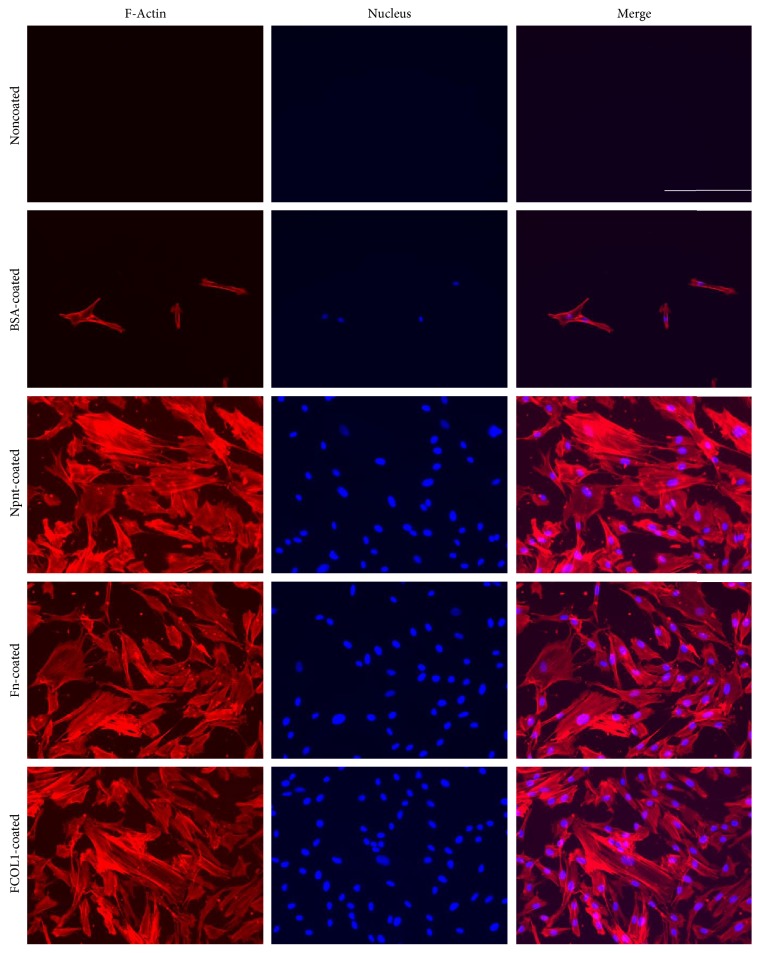
Organization of actin stress fibers. Fluorescence microscopy photographs of hDPSCs that adhered and spread on polystyrene presenting Npnt. As positive controls, cells attached to substrate(s) having adsorbed layers of Fn and FCOL1. Actin stress fibers were visualized with Alexa Fluor 568-conjugated phalloidin (red-orange), and nuclei were visualized with DAPI (blue). The scale bar in merge photo of noncoated group applies to all panels (scale bar: 200 *μ*m).

Well-developed actin stress fibers were formed and clearly observed in Npnt-, Fn-, and FCOL1-coated non-PS wells (Figure 3).

### 3.4. Cell Growth in the Absence of Serum

**Figure 4:**
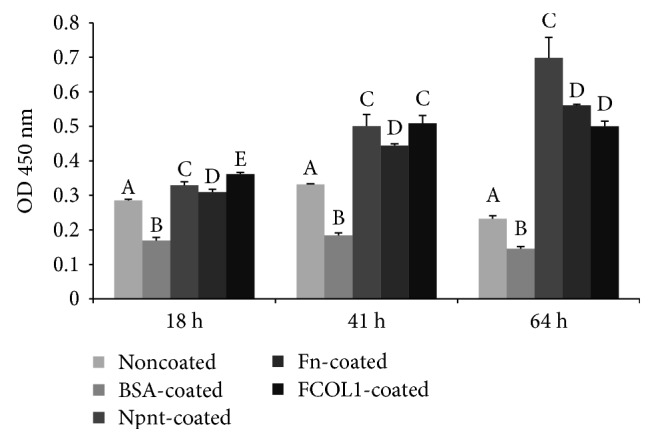
Cell proliferation in the absence of serum. hDPSCs (passage number 5) were seeded into 96-well plates (non-PS) at the concentration of 8 × 10^3^/well in FBS-free DMEM. Cell proliferation was recorded using CCK-8 reagent at 18 h, 41 h, and 64 h postinoculation. Different symbols represent significant differences in each separate time point, *p* < 0.01 by post hoc Tukey's HSD test.

The proliferation rates of hDPSCs were analyzed in response to various substrate(s) using the CCK-8 assay. The three matrix proteins (Npnt, Fn, and FCOL1) significantly promoted the proliferation of cells in the absence of serum at 18 h, and this effect persisted to 64 h (Figure 4). At 18 h, the viability of hDPSCs in Npnt (0.33 ± 0.01) was slightly lower than that of FCOL1 (0.36 ± 0.00). Forty-one hours postinoculation, the viability of the cell in Npnt (0.50 ± 0.03) was at the same level as the FCOL1 group (0.51 ± 0.02). At 64 h, cell viability of Npnt (0.70 ± 0.06) achieved the highest among the five groups, while the viability of cells in Fn was only slightly augmented; in the FCOL1 group, cell growth entered a static stage since the value at 64 h was almost unchanged as compared to that at 41 h (41 h: 0.51 ± 0.02 versus 64 h: 0.50 ± 0.02). Cells in the two negative control groups stopped to grow as illustrated by the viability number. Notably, at 64 h, viability of cells in noncoated (0.23 ± 0.01) and BSA-coated (0.15 ± 0.01) decreased as compared to their earlier time points data (noncoated: 0.29 ± 0.00 at 18 h, 0.33 ± 0.00 at 41 h; BSA-coated: 0.17 ± 0.01 at 18 h, 0.18 ± 0.01 at 41 h).

### 3.5. Peptide Inhibition Test

**Figure 5:**
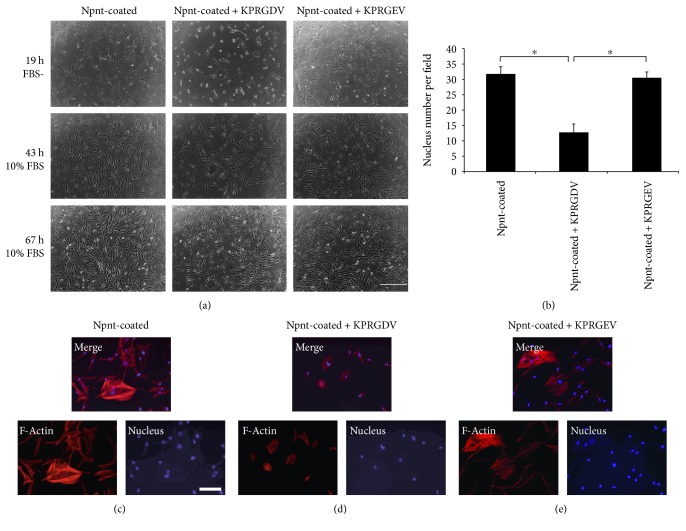
RGD was the key peptide that allowed hDPSCs to bind with Npnt. (a) hDPSCs (passage number 5) were treated with KPRGDV (1 mM in PBS) (a, middle), KPRGEV (1 mM in PBS) (a, right), or equal volume of PBS (Control) (a, left) for 10 min and were then seeded onto Npnt (10 *μ*g/mL)-coated substrate(s) (24-well plate) in serum-free DMEM, and media were changed into FBS (10%) containing DMEM after 19 h. Scrambled peptide (KPRGEV) did not inhibit cell adhesion (a, 19 h right). KPRGDV abrogated cell adhesion and spreading (a, 19 h middle). The abrogation was reversible when serum-free DMEM was replaced with 10% FBS containing DMEM (a, 43 h and 67 h) (scale bar: 400 *μ*m). (b) Nucleus number determination in four separate fields under microscopy for each group. ^∗^*p* < 0.01 by post hoc Tukey' HSD test. (c) hDPSCs were first treated with PBS (c), KPRGDV (d), and KPRGEV (e) and subsequently seeded into Npnt-coated substrate(s) in serum-free DMEM for 24 h. Fluorescence staining was conducted to visualize actin stress fibers and nucleus. Well-development actin stress fibers were observed in (c) and (e), whereas cells remain round in (d) (scale bar: 100 *μ*m).

Figure 5 shows that adhesion and spreading of hDPSCs was inhibited by KPRGDV (Figure 5(a), middle photo of the upper panel, and 5(b)); cells were successfully attached and spread on Npnt-coated surfaces in KPRGEV- or PBS-treated groups. The counting of DAPI-stained nucleus further confirmed a significant reduction of attached cell number in Npnt-RGD preincubated group, indicating adhesion of cells to the Npnt-coated substrate(s) was partly mediated by the RGD domain within Npnt (Figure 5(b)). Moreover, addition of KPRGDV hampered the spreading of hDPSCs as well (Figure 5(d)). A change of aspartic acid (D) into glutamic acid (E) completely abrogated the inhibition: cells in control peptide-KPRGEV group had no adverse effect on the parameter measured (Figures 5(a), 19 h FBS-, 5(b), and 5(e)). To test whether the inhibitory effect of RGD peptide is reversible, after 19 h of adhesion, the serum-free DMEM was replaced by the serum-containing complete medium. As shown in Figure 5(a) (middle and lower panel) that, at 43 h and 67 h, the cell adhesion and spreading in the Npnt-RGD group was recovered after incorporation of FBS, implicating this inhibitory activity of cell adhesion was reversible.

### 3.6. Real-Time RT-PCR

**Figure 6:**
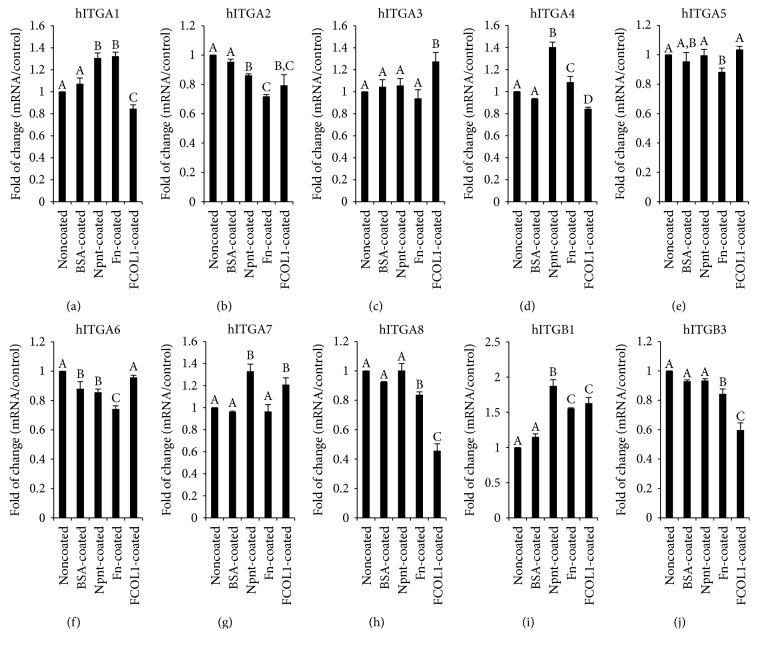
Real-time RT-PCR of integrins on day five. hDPSCs (passage number 2) were inoculated at the concentration of 1 × 10^4^/mL in 12-well plates (nontissue culture-treated polystyrene, Falcon) in DMEM supplemented with 10% FBS and penicillin/streptomycin (pen: 50 U/mL; strep: 50 *μ*g/mL) and cultured for five days. RNA was isolated at day five and reverse transcribed into cDNA. The quantitative mRNA expression of integrin *α*1 (ITGA1, (a)), *α*2 (ITGA2, (b)), *α*3 (ITGA3, (c)), *α*4 (ITGA4, (d)), *α*5 (ITGA5, (e)), *α*6 (ITGA6, (f)), *α*7 (ITGA7, (g)), *α*8 (ITGA8, (h)), *β*1 (ITGB1, (i)), and *β*3 (ITGB3, (j)) was determined by real-time RT-PCR. Results are shown as fold increase in relation to the noncoated control and represent the mean ± STD of three independent experiments. Different symbols mean significant differences in each separate panel, *p* < 0.01 (except for *p* < 0.05 between BSA-coated and Npnt-coated in (b); noncoated and Fn-coated and BSA-coated and FCOL1-coated in (d); Noncoated and Fn-coated and Npnt-coated and Fn-coated in (e); BSA-coated and FCOL1-coated in (f); BSA-coated and Fn-coated in (h); and BSA-coated and Fn-coated in (j)) by post hoc Tukey's HSD test.

**Figure 7:**
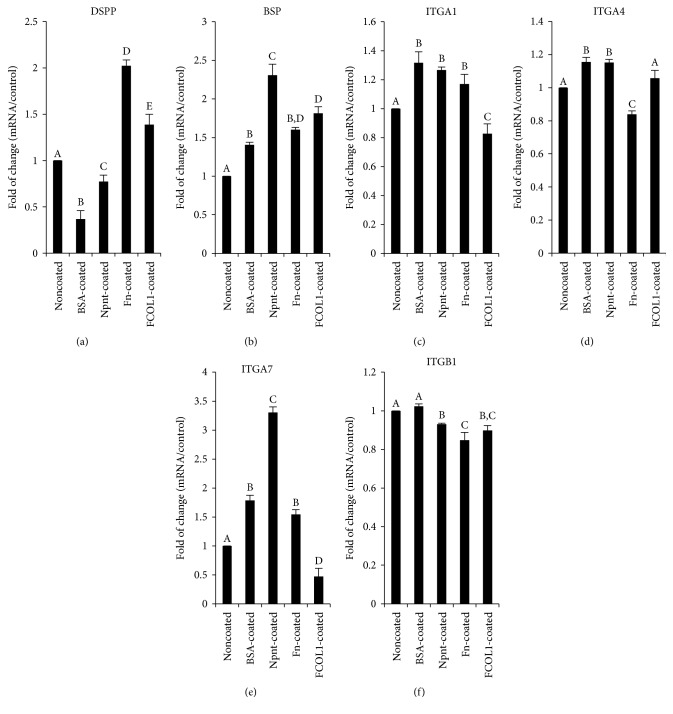
Real-time RT-PCR of DSPP, BSP, and integrins on day 28. hDPSCs (passage number 4) were cultured in different substrate(s) in DMEM supplemented with 10% FBS for five days. Mineralization reagent including *β*-GP and AA was incorporated on day five. Dexamethasone (100 nM) (D2915, Sigma) was started to be added on day nine. The total culture time was 28 days. DSPP (a), BSP (b) and ITGA1 (c), ITGA4 (d), ITGA7 (e), and ITGB1 (f) mRNA expression was investigated. Different symbols represent significant differences, *p* < 0.01 (except for *p* < 0.05 between noncoated and Npnt-coated in (a); noncoated and BSA-coated and BSA-coated and FCOL1-coated in (b); noncoated and Fn-coated and noncoated and FCOL1-coated in (c); BSA-coated and FCOL1-coated in (d); noncoated and Fn-coated in (e); and noncoated and Npnt-coated in (f)) by post hoc Tukey's HSD test.

The gene expression profile of integrin(s) at an early stage (day five) was characterized. It is shown in Figure 6 that ITGA1 and ITGB1 were simultaneously upregulated to 1.31 ± 0.04-fold (*p* < 0.01) and 1.87 ± 0.09-fold (*p* < 0.01) by Npnt, respectively. Interestingly, expression of ITGA8, the reported potent receptor for Npnt, was found to be unaltered in Npnt-coated well. ITGA2 was downregulated in the Npnt-coated group (0.86 ± 0.01-fold, *p* < 0.01). There was no change in the expression of ITGA5 in the Npnt-coated group, while in the Fn-coated group, its expression was moderately attenuated as compared to control. On day 28, the expression of hard tissue-forming markers including DSPP, BSP, and four integrins that were upregulated by Npnt on day five was assessed (Figure 7). Fn and FCOL1 significantly promoted the expression of DSPP to 2.02 ± 0.06-fold and 1.39 ± 0.11-fold, respectively, while Npnt downregulated the expression (0.77 ± 0.07). On the other hand, BSP was found to be augmented by all the three matrix proteins to a different extent (Npnt: 2.31 ± 0.14-fold; Fn: 1.60 ± 0.03-fold; and FCOL1: 1.81 ± 0.09-fold). As for the four subtypes of integrin, it was found that ITGA7 was the one that has been significantly promoted by Npnt (3.31 ± 0.10-fold); on the contrary, FCOL1 repressed its expression by 53% compared to noncoated control. Slight upregulation of ITGA1 and ITGA4 was detected in the Npnt group, while ITGB1 was moderately suppressed.

### 3.7. Alizarin Red Staining

**Figure 8:**
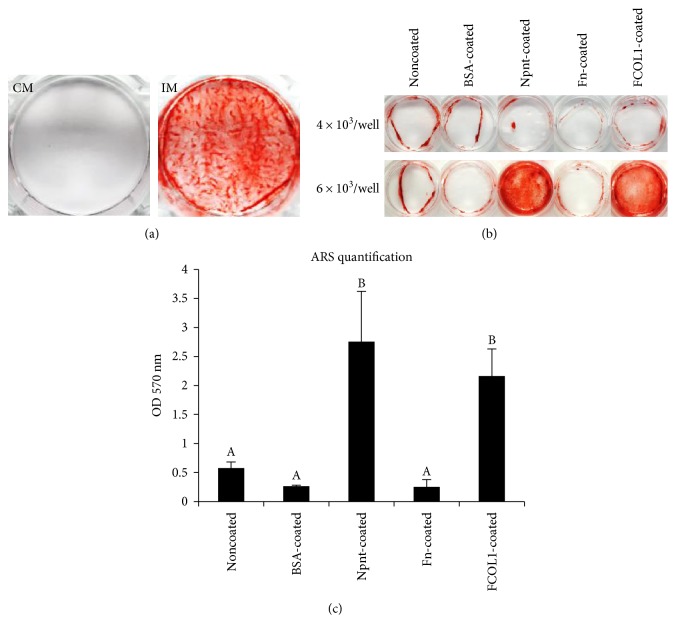
Evaluation of mineralization capacity in hDPSCs. (a) The mineralization capacity of hDPSCs was confirmed. hDPSCs (passage number 1) were seeded in 6-well plates (tissue culture-treated polystyrene, Iwaki) at the concentration of 3 × 10^4^/well in DMEM supplemented with 10% FBS and penicillin/streptomycin (50 U/mL penicillin; 50 *μ*g/mL streptomycin). Media were changed into DMEM supplemented with 5% FBS, penicillin/streptomycin and mineralization reagent (10 mM *β*-GP and 50 *μ*g/mL AA) (IM), or PBS vehicle (CM) at day four. The photo shows the alizarin red staining of cells at 23 days. (b) Alizarin red staining of hDPSCs (passage number 4) cultured on various protein-coated nontissue culture-treated polystyrene at day 30. hDPSCs were seeded to modified surfaces at the concentration of 4 × 10^3^/well (upper) or 6 × 10^3^/well (lower) in a 24-well plate (nontissue culture-treated polystyrene, Iwaki). Cells were cultured in DMEM supplemented with 10% FBS and penicillin/streptomycin. Mineralization reagent (*β*-GP and AA) was added at day 4 with 5% FBS containing DMEM. (c) Quantification of alizarin red staining for the lower panel in (b). Significant higher level of mineralization of hDPSCs was detected in Npnt-coated and FCOL1-coated substrate(s). Different symbols represent significant differences, *p* < 0.01 by post hoc Tukey's HSD test.

Figure 8(a) shows hDPSCs underwent mineralization when cultured on tissue culture polystyrene plates in osteogenic media for 19 days (total 23 days in culture), denoting that hDPSCs were able to differentiate into mineralized tissue-forming cells *in vitro*. Subsequently, it was found that mineralization occurred in cells (high seeding number 6 × 10^3^/well) cultured on Npnt- and FCOL1-coated substrate(s) (non-PS), albeit at a longer culture period (total 30 culture days and 25 days in osteogenic media). The cells grown on negative controls and Fn failed to facilitate appreciable mineralization nodules even in the presence of osteogenic media (Figures 8(b) and 8(c)).

## 4. Discussion

We used nontissue culture-treated polystyrene (non-PS), a highly hydrophobic substrate to study the roles of three matrix proteins in hDPSCs. For the surface modification of polystyrene, a variety of methods are available: physical adsorption (PA), chemical crosslinking, plasma modification, photochemical immobilization, and layer-by-layer self-assembly [11]. Among the methods listed, PA, also called physisorption, is the simplest and fastest way of achieving immobilization of a target polymer. The primary mechanism of PA lies in intermolecular interaction—the van der Waals force [12]. We hence selected the PA method because of its convenience and safety. The proteins were all prepared in solution form and immobilized by applying to non-PS at the same concentration of 10 *μ*g/mL. The coated surfaces were left dried up at room temperature. Primary antibody specific to Npnt was used to verify that the protein had undergone efficient immobilization (Figure 1). The cells were allowed to adhere to 24-well plates (non-PS) presenting Npnt, Fn, and FCOL1, simultaneously; those grown on PBS and BSA-coated wells were taken to be negative controls. Notably, a considerable number of cells started to adhere and spread as early as 1 h 30 m in the FCOL1 group: cells in FCOL1 formed membrane protrusions to adhere and attach to the underlying substratum, while those in the negative controls and Fn group remained round in shape. In the Npnt-coated group, although less in number than the FCOL1 group, cell membrane protrusions could still be observed.

Early observation of cell protrusions formed by actin polymerization in the FCOL1 and Npnt groups indicated potential positive effects adopted by these two proteins in directing differentiation of cells, which were echoed by the later ARS staining data. Eighteen hours post inoculation, all the three matrix protein ligands mediated efficient adhesion of cells and spreading to result in a flattened morphology. Therefore, for non-PS presenting the proteins at this specific concentration (10 *μ*g/mL), each protein could efficiently facilitate cell adhesion and spreading. As negative controls, PBS and BSA failed to assist efficient cell adhesion and spreading throughout the time points observed, suggesting that neither PBS nor BSA possessed cell adhesive motifs and hence did not impart cell adhesion activities. Cell number of the five groups further revealed that significant higher numbers of cells were attached in Npnt, Fn, and FCOL1 groups as compared to PBS and BSA controls (Figure 2(b)).

Next, cells were allowed to attach to substrata presenting the three types of protein and fixed to permit imaging of the cytoskeletal structures. We used phalloidin AF 568 (red fluorescence) to visualize actin stress fibers. hDPSCs had well-spread shape, with actin fibers that spanned the dimensions of cells in Npnt, Fn, and FCOL1-coated surfaces (Figure 3). However, cells cultured in PBS- or BSA-coated wells failed to generate similar cytoskeletal structure as shown in the matrix protein-coated groups. It is well-documented that the driving force for the formation of membrane protrusion is polymerization of actin filaments [13]. Remodeling of actin cytoskeleton plays a central role in a number of biological activities, especially, the importance of actin polymerization is underscored in the osteogenic commitment of stem cells, for example, osteogenic differentiation of mesenchymal stem cells was interrupted by the inhibition of actin polymerization using *Cytochalasin D* (an actin polymerization blocking reagent) [14, 15], while induction of actin polymerization in MC3T3-E1 cell by *Jasplakinolide* contributes to an enhanced mRNA expression of alkaline phosphatase (ALP) and osteocalcin (OCN) [16]. Moreover, inhibition of actin depolymerization promoted the differentiation [17]. The current results highlighted that Npnt could mediate similar cytoskeletal phenotypes as did the Fn and FCOL1, while PBS and BSA could not. Furthermore, we characterize the cell proliferation profile in the absence of serum in 96-well plates (non-PS) (Figure 4). It was shown that growth of cells seeded in PBS and BSA groups stagnated, as the absorbance reading for the three-time points in the two groups remained almost the same. In contrast, cells in the Npnt group continued to grow and reached the highest viability at 64 h as compared to the other groups. The above data shed novel light on the capacity of Npnt to support the growth of hDPSCs in the presence or even absence of serum, featuring its potential to facilitate cell attachment and growth in the exposed pulp, where the blood supply is usually compromised by aging or inflammation.

To further elucidate the specific cell adhesive motif within Npnt, we investigated whether synthetic hexapeptide KPRGDV derived from mouse Npnt (Npnt-RGD) and its mutant counterpart KPRGEV (Npnt-RGE) in soluble form could block the adhesion of hDPSCs to surface-presenting Npnt (Figure 5). We found that the Npnt-RGD could block cell adhesion and spreading to a surface presenting Npnt (Figures 5(a), 19 h FBS-, 5(b), and 5(d)), while the mutant Npnt-RGE did not (Figures 5(a), 19 h FBS-, 5(b), and 5(e)). Further, although Npnt-RGD inhibited the adhesion of cells in short-term (19 h), the presence of this peptide did not prevent cells from adhering and thriving in long-term culture (43 h and 67 h), when the media was replaced from serum-free DMEM to DMEM containing 10% FBS (Figure 5(a), 43 h and 67 h). With the advancing of technology such as chemical microchip array [18], more cell adhesion and functional peptides are being identified. Nevertheless, proteins harboring the RGD motif constitute a major recognition system for cell adhesion. The RGD motif is present in a large number of adhesive extracellular matrix proteins and recognized by over 20 known integrins [19]. The RGD-integrin binding-activity can be reproduced by short synthetic peptides containing RGD sequence, under the condition that it is stably immobilized to a substrate instead of simple addition to culture media [20]. Hence, small synthetic RGD sequence is usually utilized to test whether the adhesion of a specific type of cell to certain matrix protein is RGD-dependent. A previous work by Kulkarni et al. using soluble peptide (GRGDNP versus GRGESP) revealed that dentin matrix protein 1 (DMP1) promotes the cell attachment in MC3T3-E1 via the RGD domain [21]. Here, our results demonstrated for the first time that upon treatment by a soluble RGD peptide derived from Npnt, the adhesion and spreading of hDPSCs to Npnt was remarkably abrogated, denoting a cell adhesion activity mediated by RGD motif. Additionally, considering the inhibition of adhesion and spreading by RGD was partial, involvement of active sequences other than RGD is suggested therein. Indeed, aside from RGD, new evidence uncovered that the Phe-Glu-IIe (FEI) domain at the downstream of RGD acts synergistically with RGD to support the adhesion of the human brain neuroglioma cell (H4 cell) [22] to the Npnt-coated substratum. However, whether the FEI plays a similar role in hDPSCs awaits further scrutinization.

An ideal scaffold should be able to promote attachment and proliferation of a specific cell population needed for structural and functional restoration of the damaged tissue. The non-PS is highly hydrophobic, which is usually used for mammalian cells that grow in suspension and bacterial cell culture where attachment is not required. The above results conclude that Npnt is a hDPSC adhesive and supports hDPSC growth in either serum-containing or serum-free conditions. This potential of Npnt to initiate cell growth may yield novel approaches to recruit stem cells in situ.

Integrin, comprising *α* and *β* transmembrane glycoprotein subunits, is a complex family of cell adhesion receptors regulating a diverse array of functions. The cytoplasmic domains of integrin connect to the actin cytoskeleton. Accumulating evidences have shown that the cross talk between integrin and environmental clues such as extracellular matrix protein is important in mediating cell proliferation and differentiation [23]. In an effort to clarify gene expression profile of ten types of integrin (eight alpha integrins and two beta integrins), real-time RT-PCR was carried out at an early stage (day five) after cell inoculation. The results highlighted that Npnt triggered the upregulation of ITGA1 (1.31 ± 0.04-fold), ITGA4 (1.40 ± 0.05-fold), ITGA7 (1.33 ± 0.07-fold), and ITGB1 (1.87 ± 0.09-fold). As a ubiquitously expressed integrin and common partner to almost all the alpha integrins, *β*1 integrin (ITGB1 or CD29) is the primary plasma membrane receptor transmitting cues from the extracellular matrix to intracellular signaling pathways; here, the upregulation of ITGB1 correlates well with our previous data using MDPC-23 cell line [10]. Ozeki et al. reported enhanced ITGA1 expression leads to the differentiation of human skeletal muscle stem cells (hSMSCs) into odontoblasts evidenced by upregulation of dentin sialophosphoprotein (DSPP), dentin sialoprotein (DSP), and alkaline phosphatase (ALP) [24]. In addition, dentin phosphoprotein- (DPP-) coated polystyrene can augment the expression of ITGA4 in rat preodontoblast cell line (T4-4) [25]. Another study showed that ITGA7 positive hSMSCs have the potential of differentiation into odontoblasts under appropriate inductive conditions (retinoic acid and bone morphogenetic protein-4 (BMP4)) [26]. The findings in the present experiment suggest that *α*1*β*1, *α*4*β*1, and *α*7*β*1 might be involved during early stages of Npnt-mediated adhesion and intracellular signaling. In contrast, there was no elevation in the expression of ITGA8 as measured by quantitative RT-PCR in the Npnt-coated group. The unaffected expression of ITGA8 in the Npnt group suggests either a compensatory effect from other alpha integrins, such as ITGA1, ITGA4, and ITGA7 forming heterodimers with integrin *β*1 or/and an earlier role of ITGA8 that was not detected because of its late downregulation. Additionally, mild attenuation of ITGA2 and ITGA6 expression was observed in the Npnt-treated group. Except for the elevation of ITGA3 and ITGA7, FCOL1 downregulates the rest alpha integrins (ITGA1, ITGA2, ITGA4, and ITGA8) without impacting ITGA5 and ITGA6. Notably, the expression ITGA8 (0.46 ± 0.05-fold, *p* < 0.01) was found to be remarkably reduced under the influence of FCOL1. Fn (1.55 ± 0.01-fold, *p* < 0.01) and FCOL1 (1.63 ± 0.08-fold, *p* < 0.01) both enhanced the expression of ITGB1 at a level of statistical significance, while there was no difference between the two groups. With regard to ITGB3, it was found that its mRNA expression remains constant in noncoated, BSA-coated, and Npnt-coated groups and was inhibited by both Fn and FCOL1. The data demonstrated Npnt selectively upregulated mRNA expression of ITGA1, ITGA4, and ITGA7. However, it remains to be clarified which specific integrin receptor (*α*1*β*1, *α*4*β*1, or *α*7*β*1) plays a predominant role in mediating the adhesion and differentiation of hDPSCs. To answer this question, inhibition experiment is necessary using antibodies against *α*1*β*1, *α*4*β*1, and *α*7*β*1 in the future study.

Further, the late-stage gene expression analysis in Figure 7 revealed that Npnt tended to direct the hDPSCs' differentiation toward osteoblastic lineage instead of odontoblastic lineage, as the DSPP, a well-established odontoblast differentiation marker [27], was moderately suppressed by Npnt, but BSP, a widely recognized osteoblast marker, [28] was markedly enhanced by Npnt compared to the other groups. The result correlates well with a previous work from Kahai et al. [29], indicating that Npnt induces the differentiation of hDPSCs into osteoblast rather than odontoblast. Nevertheless, Npnt still holds sound potential for its application in the pulp capping treatment, as a coating of Npnt significantly augments the matrix mineralization of hDPSCs. In addition, ITGA7 was found to be remarkably increased by Npnt as much as threefold more than the noncoated control, denoting that the ITGA7 may be required in the interaction between Npnt and hDPSCs at the late stage of osteoblastic differentiation.

To examine whether a coating of polystyrene by the matrix proteins might cause an enhanced mineralization, the *in vitro* mineralization capabilities of hDPSCs in various substrate(s) were monitored. As expected, the induction of differentiation of cells in Npnt and FCOL1 resulted in the appearance of mineralized nodules revealed by alizarin red staining at day 30. Moreover, we observed that a low initial seeding number fail to initiate the mineralization after culturing for the same period of time, be it noncoated or protein-coated surfaces, denoting a sufficient number of cells (in the present work: 6 × 10^3^/mL in 24-well plates) are required to ensure the onset of appreciable late-stage calcific deposition. In terms of mineralization activity, a comparison of Npnt, Fn, and FCOL1 revealed that both Npnt and FCOL1 were promising in eliciting intensive calcific deposition. The finding suggested Npnt assumes a comparable mineralization-inducing capacity with FCOL1, which is a well-documented tissue engineering material [30, 31]. Interestingly, although the RGD-containing Fn supported hDPSC proliferation, cells grown on it did not undergo an appreciable mineralization as did Npnt and FCOL1. An earlier work demonstrated that Fn promotes the mineralization, albeit to a lesser extent than COL1, in bovine vascular cells [32]. A possible explanation for the observed phenomena might be attributable to a different type of cell used. Moreover, except RGD, there are other cell adhesive motifs in the protein, namely, PHSRN. The interaction of Fn with cells was not merely via RGD but from a synergistic action of RGD, PHSRN, and other potential motifs. Further, there are various adhesive proteins in FBS and those secreted by cells; the coated proteins did not exert their influence in isolation: cells exposed to the immobilized proteins were interacting against a background of various native integrin-binding proteins. The alizarin red staining data revealed that an accelerated cell adhesion and proliferation does not necessarily leads to the final enhancement of mineralization.

Based on the findings, it is suggested that Npnt possesses comparable capacity to FCOL1 in promoting proliferation and eliciting intensive mineralization in hDPSCs. However, with regard to the cost-effectiveness, FCOL1 stands a better chance of future application, as the price for FCOL1 is much cheaper than Npnt. Despite this limitation, the present experiment has clarified a novel role for Npnt in modulating hDPSC integrin expression and altering their interaction with the microenvironment, leading to mineralization.

## 5. Conclusion

Taken together, the results demonstrated that Npnt facilitates hDPSC adhesion and spreading to hydrophobic nontissue culture-treated polystyrene. The adhesion and spreading of hDPSCs to Npnt was partially mediated by RGD motif. Moreover, Npnt acts as a bioactive signal to initiate the upregulation of ITGA1, ITGA4, ITGA7, and ITGB1 as early as day five. Further, the mRNA expression of DSPP was slightly downregulated by Npnt, while BSP was significantly enhanced. Finally, late-stage mineralization was significantly enhanced in cells cultured in Npnt-coated and FCOL1-coated wells as compared to negative controls.

## Supplementary Material

Supplementary file 1: Certificate of analysis for hDPSCs from LONZA company.

## Figures and Tables

**Table 1:** Primer sequence, fragment size, and annealing temperature.

Gene name	Forward	Backward	Fragment size (bp)	Annealing temperature (°C)
hGAPDH (NM_001289746.1)	CACTAGGCGCTCACTGTTCTCT	CGTTCTCAGCCTTGACGGT	250	66
hDSPP (NM_014208.3)	TGCTGGCCTGGATAATTCCG	CTCCTGGCCCTTGCTGTTAT	136	66
hBSP (NM_004967.3)	AAGGGCACCTCGAAGACAAC	CCCTCGTATTCAACGGTGGT	119	62.8
hITGA1 (NM_181501.1)	CTCACTGTTGTTCTACGCTGC	ACGACTTGAAATGTGGGGCT	419	59.9
hITGA2 (NM_002203.3)	GTGGCTTTCCTGAGAACCGA	GAAGCTGGCTGAGAGCTGAA	278	62.8
hITGA3 (NM_002204.3)	ATGGCAAGTGGCTGCTGTAT	GCACTCTAGCCACACACAGT	272	59.9
hITGA4 (NM_000885.5)	AATCCCGGGGCGATTTACAG	TCCAGCTTGACATGATGCAAAA	354	59.9
hITGA5 (NM_002205.4)	CCCTCATCTCCGGGACACTA	ATCCAACTCCAGGCCCTTTG	397	56.3
hITGA6 (NM_000210.3)	CTCGCTGGGATCTTGATGCT	TCAGATGGCTGAGCATGGAT	128	59.9
hITGA7 (NM_002206.2)	GGAAGACCGACAGCAGTTCA	ATCTTGATGCGACACCAGCA	264	59.9
hITGA8 (NM_003638.2)	GCCTATGCCGAGTTCTCTCC	CCCAGTAAACTCCCCAGCAG	297	59.9
hITGB1 (NM_002211.3)	GCCGCGCGGAAAAGATGAAT	TGCTGTTCCTTTGCTACGGT	323	59.9
hITGB3 (NM_000212.2)	GAAGCAGAGTGTGTCACGGA	ACATGACACTGCCCGTCATT	201	59.9
