# Supplementary material for: The Role of Nephronectin on Proliferation and Differentiation in Human Dental Pulp Stem Cells
Source: Stem Cells Int. 2017 Nov 19;2017:2546261. doi: 10.1155/2017/2546261 (PMC5735320; doi:10.1155/2017/2546261)
Supplement: Supplementary file 1 — Certificate of analysis for hDPSCs from LONZA company. [file 2546261.f1.pdf]

Lonza Walkersville Inc.
8830 Biggs Ford Road
Walkersville, MD 21793 8415
Tel (301) 898 7025
Fax (301) 845 4024

Lonza

CERTIFICATE OF ANALYSIS

Product Code: PT-5025
Product: Human Dental Pulp Stem Cells 1M

Lot Number: 0000361427
Manufacture Date: 21-Mar-2013

TEST (Method)	SPECIFICATIONS Min.	Max.	Results
Tissue Acquisition Number	***	***	25885
DONOR CHARACTERISTICS			
Age	***	***	16 Y
Sex	***	***	MALE
Race	***	***	UNKNOWN
VIRUS TESTING			
HIV Test	***	***	Not Detected
HBV Test	***	***	Not Detected
HCV Test	***	***	Not Detected
MICROBIAL TESTING			
Sterility Test	***	***	Negative
Mycoplasma	***	***	Negative
CELL PERFORMANCE TESTING			
Viability	>=70%	***	86 %
Cell Count (cells/vial)	>=1x10E+6	***	1470000
Total Population Doublings	>=10	***	13
Seeding Efficiency	>=25%	999	92 %
CD34, CD45, & CD133	***	<=10%	< 10.0 %
CD105, CD166, CD29, CD90, & CD73	>=90%	***	> 90.0 %

These cells were isolated from donated human tissue after obtaining permission for research use by informed consent or legal authorization. This product is for research use only. Details concerning the use of our cell and media products can be downloaded from our website at www.lonza.com/cell-protocols.

This lot has been reviewed by Quality Assurance in compliance with requirements of Lonza's Quality System.
This document was generated from a validated Part 11-compliant electronic system and thus handwritten signatures are not required.
